# Pediatric Gastroenterology and Hepatology in Italy before and after the COVID-19: Lessons learned and management changes by SIGENP

**DOI:** 10.1186/s13052-023-01418-7

**Published:** 2023-01-26

**Authors:** Valeria Dipasquale, Marco Deganello Saccomani, Angelo Di Giorgio, Salvatore Oliva, Silvia Salvatore, Caterina Strisciuglio, Renato Tambucci, Paolo Lionetti, Claudio Romano

**Affiliations:** 1 Pediatric Gastroenterology and Cystic Fibrosis Unit, Department of Human Pathology in Adulthood and Childhood “G. Barresi”, University of Messina, Messina, Italy; 2 Department of Pediatrics, Woman’s & Children’s University Hospital of Verona, 37126 Verona, Italy; 3 Pediatric Hepatology, Gastroenterology and Transplantation, Hospital Papa Giovanni XXIII, Bergamo, Italy; 4 Pediatric Gastroenterology and Liver Unit, Maternal and Child Health Department, Sapienza University of Rome, Rome, Italy; 5 Department of Pediatrics, “F. Del Ponte” Hospital, University of Insubria, Varese, Italy; 6 Department of Woman, Child and General and Specialistic Surgery, University of Campania “Vanvitelli”, Naples, Italy; 7 Digestive Endoscopy Unit, Department of Gastroenterology, Hepatology and Nutrition, Bambino Gesù Children’s Hospital, Rome, Italy; 8 Department NEUROFARBA, University of Florence, Gastroenterology and Nutrition Unit, Meyer Children’s Hospital, Florence, Italy

**Keywords:** Anti-TNF-α, COVID-19, Digestive endoscopy, Hepatitis, Inflammatory bowel disease, Liver disease, Pediatrics, SARS-CoV-2, SIGENP, Telemedicine

## Abstract

﻿Around the world, the 2019 Coronavirus disease (COVID-19), caused by the severe acute respiratory syndrome coronavirus 2 (SARS-CoV-2), has raised serious public health problems and major medical challenges. The Italian Society of Pediatric Gastroenterology, Hepatology and Nutrition (SIGENP) published several papers on ﻿the impact of COVID-19 on the current management, diagnosis, and treatment of acute and chronic gastrointestinal, hepatic, immune-mediated, and functional disorders. The present article summarizes the most relevant SIGENP reports and consensus during and after the peak of the COVID-19 outbreak, including the diagnosis and treatment of inflammatory bowel disease (IBD), indications and timing of digestive endoscopy, and insights into the novel hepatitis.

## Introduction

The severe acute respiratory syndrome coronavirus 2 (SARS-CoV-2) resulted in the 2019 Coronavirus disease (COVID-19) and pandemic [1]. After the first outbreak, children showed similar rates of SARS-CoV-2 infection as adults, although the pediatric COVID-19 is generally milder or silent [2, 3], severe cases have been reported in selected subjects [4]. Moreover, the diagnostic work-up, management of children with complexities, and healthcare access have been impacted by the widespread lockdown and restrictions imposed by the pandemic [5]. The Italian Society of Pediatric Gastroenterology, Hepatology and Nutrition (SIGENP) published several papers on ﻿the impact of COVID-19 on the diagnosis and treatment of chronic gastrointestinal disorders and immune-mediated diseases, particularly inflammatory bowel disease (IBD), which has been the source of major concern in the last three years. This article reports the evidence and recommendations developed by SIGENP during and after the peak of the COVID-19 outbreak.

### Inflammatory bowel disease

Children with IBD are at high risk of infection, particularly opportunistic infections, due to multifactorial immunodeficiency [6, 7]. Immunological dysregulations that commonly underlie IBD, immunodeficiencies, poor nutritional status, and, most importantly, the higher need for early and/or combined immunosuppressive and biologic therapies in comparison to adult patients predispose patients to infections [6, 7]. Autoimmune dysregulation and the use of immunosuppressants and/or biologics were postulated as risk factors for a more severe course of COVID-19 in these individuals.

[8]. ﻿However, temporal trends in reporting COVID-19 in patients with IBD aligned with global epidemiological patterns [9]. From March 2020 to July 2021, 6404 cases of COVID-19 were reported in IBD patients across 73 countries in the Surveillance Epidemiology of Coronavirus Under Research Exclusion (SECURE-IBD) database [10]. Furthermore, despite the use of immunosuppressants and/or biologics, both the SECURE-IBD database and the European Society for Pediatric Gastroenterology, Hepatology, and Nutrition (ESPGHAN) IBD Porto group reported mostly mild SARS-CoV-2 infections in pediatric IBD patients without the need for hospitalization [8, 10]. Likewise, a multicenter, cross-sectional survey of IBD children in Lombardy during the first lockdown showed that 8% of IBD children complained of mild symptoms indicative of COVID-19, with no need of hospitalization or change in IBD treatment, including biologics [11]. Notably, there was no substantial difference in IBD diagnosis and flares from the prior year [11].

#### Diagnosis of IBD in the COVID-19 era

During the COVID-19 pandemic, elective visits and endoscopies were rescheduled and/or postponed, potentially having a clinical and psychological influence on disease diagnosis and progression [12]. In 2020, the SIGENP IBD Working Group conducted a multicenter, retrospective cohort study involving 21 Italian IBD referral centers and a total of 2291 pediatric IBD patients [13]. The objective of the study was to compare the clinical activities undertaken before and after the COVID-19 pandemic lockdown in the management of pediatric IBD patients across the Italian territory. The main results were a significant reduction in hospital admissions, including inpatients, outpatients, and transition visits (from 55.9% to 26.3% before and during lockdown) [13]. These changes in IBD care were consistent with both the Italian health system's indications to postpone non-urgent activities and the guidelines released by international adult and pediatric IBD societies [8, 14, 15]. Following the peak of the COVID-19 pandemic, the SIGENP IBD Working Group produced a position paper and recommendations for IBD diagnostic workup (Table 1) [16].

**Table 1:** ﻿Hospital access and diagnostic procedures for IBD after the peak of the COVID-19 pandemic (adapted from the SIGENP position paper) [16]

Topic	Recommendation
﻿Hospital access	Limited; priority levels based on the spread of COVID-19; activation of telemedicine initiatives
﻿Endoscopic procedures	Maintained in all of the following situations: • Mild to moderate flare-ups with abnormal blood tests • Symptoms of mild subacute obstruction already confirmed by imaging • New diagnosis • Monitor for postoperative recurrence within 1 year of surgery if patient is symptomatic or has elevated calprotectin or altered blood tests • Symptomatic pouchitis and altered blood tests • Reevaluate symptomatic patients with abnormal fecal calprotectin or blood tests after 6 months of biological therapy
﻿Nasopharyngeal swab test before endoscopy	Recommended when performing endoscopies under deep sedation and/or general anesthesia
Telemedicine	﻿Implementation/reinforcement recommended

COVID-19, 2019 Coronavirus disease

The SIGENP IBD Working Group also focused on the use and implementation of telemedicine services, which have emerged as relevant healthcare resources over the last few years [17–19]. Subjects with stable disease on maintenance therapy could adequately be managed by a scheduled program of telemedicine [17]. The sudden SARS-CoV-2 pandemic represented an urgent need to test and implement telemedicine services, even for pediatric IBD [20, 21]. The results of the SIGENP multicenter cohort study showed that specific telemedicine services for children with IBD were activated in 52.3% of the participating centers [13]. The study also highlighted how telemedicine might increase patients’ quality of life and decrease hospitalization rates and healthcare costs.

During the peak of COVID-19, diagnostic endoscopies for suspected IBD fell by 15.5%, while procedures to assess mucosal healing decreased by 48.3%, according to a recent survey conducted by the SIGENP Endoscopy Working Group (see below) [22]. Nevertheless, the SIGENP IBD Working Group recommended not deferring endoscopic procedures in cases of confirmation of new diagnoses, severe acute flares, or the occurrence of complications (Table 1) [16]. Assessment and screening for signs of infection, travel history, and exposure to potentially infected patients should be promptly investigated upon hospital admission [5, 16]. Patients should undergo a SARS-CoV-2 nasopharyngeal swab test 24–48 h before endoscopy under deep sedation and/or general anesthesia [16], as is common in children. More details on pediatric digestive endoscopy are reported and discussed further in the “Digestive endoscopy” section.

#### Treatment of IBD in the COVID-19 era

﻿During the outbreak, healthcare providers and IBD experts debated whether IBD treatment should be discontinued for mild or moderate COVID-19 cases [8, 23], particularly in children living in an endemic area and receiving biologics and immune suppressive agents. The steroid therapy caused particular anxiety, since studies have shown that steroids are more likely to cause infections than anti-tumor necrosis factor (TNF)-α or other immunomodulators [6, 7]. The SIGENP-IBD Working Group made recommendations for the pharmacological management of IBD in the post-COVID-19 peak periods (Table 2) [16].

**Table 2:** Recommendations for the pharmacological management of IBD ﻿after the peak of the COVID-19 pandemic (adapted from the SIGENP position paper) [16]

Topic	Recommended
Steroids for induction	Yes (accelerated steroid weaning if clinically indicated)
Suspension of immunomodulators	
▪As monotherapy	No
▪As combination therapy	No
Suspension of biologics	
▪As monotherapy	No
▪As combination therapy	No
Start of immunosuppressants or anti-TNF-α	Yes
Intervals and doses of anti-TNF-α	Regular
RT-PCR/serological screening for SARS-CoV-2 before starting anti-TNF-α therapy	No
Vedolizumab and ustekinumab use	Yes (not contraindicated)

TNF-α, tumor necrosis factor-α; RT-PCR, real time-polymerase chain reaction; SARS-CoV-2, severe acute respiratory syndrome coronavirus 2

Apart from eventually modifying the steroid treatment, there is no increased risk of SARS-CoV-2 infection in adults and children with IBD compared to non-IBD patients [8, 11]. Some warnings have been reported about the use of prednisone at a dose of 20 mg/day in adults at increased risk of SARS-CoV-2 infection [24]. Brenner et al. [25] published the first data from the SECURE-IBD ​​registry, which included both pediatric and adult IBD patients. The analysis included 525 cases of patients with IBD and COVID-19, with a median age of 41 years. In the multivariate analysis, steroid therapy was the most significant factor in the development of severe COVID-19. However, there are no data on low-dose or short-term use of steroids. A steroid taper should always be undertaken under close medical supervision [16], and the risk/benefit of prednisone use should be assessed on a case-by-case basis, particularly in children with acute severe colitis (ASC) or significant flares of IBD.

﻿To date, therapies targeting TNF-α have not been associated with adverse outcomes in IBD patients with COVID-19 [25]. Anti-TNF-α had an immediate beneficial effect on the virus-induced cytokine storm in both children and adults with active Crohn's disease and COVID-19 [26]. Anti-TNF-α agents are likely to have a therapeutic effect by: (i) lowering levels of TNF-α, which are elevated in patients with severe COVID-19 infection; and (ii) downregulating transmembrane angiotensin converting enzyme-2 (ACE-2) receptors, which have been identified as the virus's anchor site [25, 26]. Early consideration of biologics such as anti-TNF-α has been suggested for the treatment of a newly reported clinical entity called Pediatric inflammatory multisystem syndrome-temporally associated with SARS-CoV-2 (PIMS-TS) or Pediatric Multisystem Inflammatory Syndrome (MIS-C) [27, 28].

### Digestive endoscopy

Elective endoscopies have been postponed or cancelled across Europe during the COVID-19 outbreak [29] since these procedures were deemed to be at high viral transmission risk. Upper digestive tract endoscopy can cause airborne transmission by inducing coughing, gagging, and retching, whereas colonoscopy can result in the passage of flatus and pathogen-carrying liquid stools. In December 2020, the SIGENP Endoscopy Working Group conducted a web-based survey among 24 Italian pediatric centers that perform digestive endoscopies [22]. The purpose of this study was to assess the impact of COVID-19 on procedural volumes, indications, healthcare provider safety perceptions, and waiting times for pediatric endoscopies. Between 2019 and 2020, there was a 37.2% decrease in digestive endoscopies, with a substantial decrease in the median number of procedures (111 vs 57, *p* < 0.001) [22]. The median number of procedures performed either for new diagnosis or follow-up declined significantly in 2020 (63 vs 36, *p* < 0.001 and 42 vs 21, *p* < 0.001, respectively) [22]. While endoscopies for new diagnoses of celiac disease, functional gastrointestinal disorders (FGIDs), suspected Helicobacter pylori infection, eosinophilic gastroenteropathy, or to exclude FGIDs were significantly reduced, only a minor drop was noted for IBD new diagnoses [22]. In particular, the total number of endoscopies scheduled for suspected celiac disease at the 24 participating centers declined from 621 to 279 (a 55.1% decrease). However, the updated ESPGHAN recommendation to examine the feasibility of a biopsy-free technique for the identification of celiac disease in a restricted group of children [30] may have contributed to a decline in the use of endoscopy for celiac disease diagnosis. Moreover, due to the lack of available endoscopic slots during the pandemic, some sites decided to consider a lower antibody threshold to make a biopsy-free diagnosis [31]. A modification of the indications for digestive endoscopy procedures is warranted in the context of endoscopic restriction. Given pandemic-related resource limitations, patient triage is a priority to balance the need to curtail viral spread with the ramifications of potentially delayed diagnosis, particularly for endoscopic procedures. SIGENP proposed throughout its network triage protocols to help pediatric gastroenterologists in indicating endoscopic procedures during the pandemic (available at: https://sigenp.org/wp-content/uploads/2020/10/Endoscopia_Digestiva_COVID19.pdf). The North American guidelines outlining a risk stratification of pediatric endoscopic procedures during the pandemic also tried to address this need [32]. During COVID-19, SIGENP proposed considering avoiding intestinal biopsy for diagnostic confirmation in case of symptomatic patient with positive IgA anti-endomysial antibodies and IgA tissue transglutaminase antibodies > 7.5 × and < 10 × cut-off (https://sigenp.org/wp-content/uploads/2020/10/MC_COVID.pdf).

### Functional gastrointestinal disorders

FGIDs are a group of common gastrointestinal symptoms, such as infantile regurgitations, colic, cyclic vomiting, abdominal pain, dyspepsia, diarrhea, and constipation, that affect 10–30% of Italian infants and children [33, 34]. FGIDs are a complex phenomenon determined by genetic and environmental factors, including early life and adverse events, stress, psychological disturbances, pain perception and coping, microbiota, nutrition, drugs, and infections [35, 36]. In 2015, a SIGENP multicenter study recruiting children with different enteric infections revealed a significantly higher risk (RR = 1.9) of abdominal pain-related FGIDs in the 6-month follow-up compared to controls [37].

A systematic review of gastrointestinal manifestations in children presenting COVID-19 early pointed out that, in Italy as well as over the world, diarrhea, vomiting, and abdominal pain are common in children with COVID-19, and, in some cases, they may represent the first and sole clinical manifestation of SARS-CoV-2 infection, with gut persistence of the virus longer than in the upper respiratory tract [38]. However, only limited data are currently available on the impact of COVID-19 pandemic on the rate and outcome of FGIDs in children. A multicenter international observational study conducted between April and July 2020 interviewed 356 children with a previous diagnosis of FGIDs [39]. Symptomatic improvement was reported after 4 months of follow-up, as were satisfactory QoL and anxiety scores, suggesting positive effects of school closure and increased parental attention [39]. Conversely, data from an Italian school-based survey of high school students involving 407 adolescents who completed the pediatric Rome III questionnaire during the COVID-19 virus outbreak showed a prevalence of FGIDs higher than previously reported. The most marked increase was shown in the functional abdominal pain disorders, while interestingly, the prevalence of functional constipation was similar [40]. These observations may be explained by the well-known link between psychological disorders and FGIDs, consistent with the deteriorated anxiety and depression levels after the COVID-19 pandemic reported by adolescents [41]. An increasing body of evidence has associated the COVID-19 pandemic with anxiety and altered psychological functioning that may predispose to FGIDs. In an adult cohort American study, during a 6-month follow-up, 29% of subjects reported gastrointestinal symptoms they perceived to be related to COVID-19: 16% heartburn, 11% constipation, 9.6% diarrhea, 9.4% abdominal pain, and 7.1% nausea/vomiting, with a strong association with mental health symptoms [42].

Studies are underway to assess the prevalence of FGIDs in a cohort of neonates born in Italy during the COVID pandemic and to investigate the influence of stress and SARS-CoV-2 infections in pediatric age groups.

### Liver disease

﻿COVID-19-related liver injury presents with a mild elevation of transaminases, although its clinical significance is unclear [43]. Studies reported that children with chronic liver disease (*n* = 369 patients), autoimmune liver disease (*n* = 47 patients), and liver transplanted patients (*n* = 155 patients) maintained a good health status during the SARS-CoV-2 outbreak in Northern Italy [44–46]. These results suggest that children with chronic liver disease do not have an increased risk for severe disease course of SARS-CoV-2 infection with little or no liver dysfunction. As a result, maintaining normal standards of care while adhering to national COVID-19 recommendations is critical, as is maintaining immunosuppressive medicine to prevent recurrence or rejection.

Following the discovery of a cluster of pediatric cases of hepatitis of unknown etiology (called “novel pediatric hepatitis of unknown etiology” or “novel hepatitis”) identified in Scotland in March 2022 [47], the World Health Organization issued an outbreak alert, and over 1010 probable cases were reported [48]. A recently published systematic review (April 1, 2021-August 30, 2022) revealed 22 case series and case–control studies documenting 1643 cases, with 120 children (7.3%) having liver transplants and 24 deaths (1.5%) [48]. SARS-CoV-2 has been implicated; however, results for both serological testing and testing of explant or biopsy liver for both adenovirus and SARS-CoV-2 are inconclusive. A potential unifying theory regarding the role of SARS-CoV-2 involves superantigens [49]. A sequence motif on the SARS-CoV-2 spike protein was discovered to be structurally and sequentially identical to the recognized superantigen staphylococcal enterotoxin B [50]. Similarly, the SARS-CoV-2 spike protein may be able to generate broad, nonspecific T-cell activation. The SIGENP Liver Working Group conducted survey research in Italy from January to May 2022, comprising all of the major pediatric liver units [51]. Forty-four cases of novel hepatitis were reported by 27 Italian pediatric centers, including four pediatric liver transplant centers. This survey ﻿suggested a weak link between SARS-CoV-2 or adenovirus infection and the novel hepatitis. In fact, infectious organisms were found in 61.8% of the cases and SARS-CoV-2 was found in four cases [51]. Furthermore, only four individuals were vaccinated against SARS-CoV-2, showing that the vaccination played no role in the disease's emergence.

## Conclusions

COVID-19 has had an impact on patients with chronic diseases, including gastrointestinal diseases. This study summarizes the most relevant SIGENP reports and the consensus reached during and immediately after the COVID-19 pandemic in Italy. The main purpose is the need to optimize and improve the management of pediatric gastrointestinal diseases in the post-COVID-19 era. Professionals should take advantage of it by eliminating some useless steps, improving and updating old patterns, and optimizing new resources. The strength of this study comes from the presence of a summary of the recommendations from experts in a scientific society. Even after the SARS-CoV-2 pandemic has peaked, pediatric IBD management should be based on a balance between IBD activity and COVID-19 severity. After the benefits and risks have been discussed with each patient, the decision to discontinue IBD therapy should be made on an individual basis. Whenever possible, telemedicine follow-up should be initiated and/or implemented to improve follow-up options for children with stable diseases. The timing and indications for digestive endoscopy have been adjusted during the outbreak, with most non-urgent activities being postponed.

The occurrence of functional gastrointestinal disorders related to COVID-19 and pandemic is still uncertain and is a matter of current research. The novel hepatitis has raised concern in the medical community, but the link to SARS-CoV-2 infection remains unclear. The management of acute and chronic gastrointestinal diseases calls for prompt updates to keep up with the COVID-19 surges.

## Data Availability

Not applicable.

## Abbreviations

ACE-2: Angiotensin converting enzyme-2
ASC: Acute severe colitis
COVID-19: 2019 Coronavirus disease
ESPGHAN: European Society for Pediatric Gastroenterology, Hepatology, and Nutrition
FGIDs: Functional gastrointestinal disorders
IBD: Inflammatory bowel disease
MIS-C: Multisystem inflammatory syndrome in children
PIMS-TS: Pediatric Inflammatory Multisystem Syndrome-temporally associated with SARS-CoV-2
RT-PCR: Real time-polymerase chain reaction
SARS-CoV-2: Severe acute respiratory syndrome coronavirus 2
SECURE-IBD: Surveillance Epidemiology of Coronavirus Under Research Exclusion
SIGENP: Italian Society of Pediatric Gastroenterology, Hepatology and Nutrition
TNF-α: Tumor necrosis factor-α
